# Exercise-Induced Short-Chain Fatty Acids: A Novel Therapeutic Target in Type 2 Diabetes Mellitus with Sarcopenia

**DOI:** 10.14336/AD.2025.0670

**Published:** 2026-06-19

**Authors:** Fan Shi, Jun Chen

**Affiliations:** ^1^Physical Education Institute, Hubei University, Wuhan 430079, China.; ^2^School of Health Sciences and Physical Education, Macao Polytechnic University, Macao 999078, China.

**Keywords:** type 2 diabetes mellitus, sarcopenia, exercise, short-chain fatty acids, gut microbiota

## Abstract

Type 2 diabetes mellitus accompanied by sarcopenia is an emerging clinical challenge in aging populations, characterized by coexisting metabolic dysfunction and the progressive loss of skeletal muscle mass and function. This comorbidity substantially elevates the risk of frailty, functional impairment, and poor clinical outcomes, highlighting the urgent need for targeted therapeutic interventions. Growing evidence suggests that gut microbiota dysbiosis contributes to the pathogenesis of both Type 2 diabetes mellitus and sarcopenia through mechanisms such as chronic inflammation, insulin resistance, and mitochondrial and autophagic dysfunction. Among gut-derived metabolites, short-chain fatty acids exert anti-inflammatory and insulin-sensitizing effects and also promote muscle metabolism and mitochondrial function. Notably, exercise increases the abundance of short-chain fatty acid-producing bacteria, thereby elevating circulating short-chain fatty acid levels and contributing to improved glucose homeostasis and skeletal muscle function. This review summarizes the effects of various exercise modalities on short-chain fatty acid production and explores the mechanisms by which short-chain fatty acids mediate the benefits of exercise in Type 2 diabetes mellitus complicated by sarcopenia, emphasizing their potential as novel therapeutic targets for integrated disease management.

## 1. Introduction

Diabetes mellitus is a chronic metabolic disorder characterized by persistent hyperglycemia and has emerged as a major global public health concern in the 21st century. According to the International Diabetes Federation, the global prevalence of diabetes was 537 million in 2021 and is projected to rise to 783 million by 2045, with type 2 diabetes mellitus (T2DM) accounting for approximately 90% of all cases [1]. Sarcopenia is a progressive loss of skeletal muscle mass and function, broadly classified into primary sarcopenia, which is predominantly age-related, and secondary sarcopenia, which arises due to chronic diseases such as cancer, malnutrition, or metabolic disorders including T2DM. In the context of T2DM, secondary sarcopenia is increasingly recognized as a frequent and clinically significant comorbidity [2]. The interplay between insulin resistance (IR), chronic systemic inflammation, and reduced physical activity places individuals with T2DM at a significantly higher risk of developing sarcopenia [3]. The global prevalence of sarcopenia among individuals with T2DM is reported to be approximately 18%, with varying rates across different regions [4]. In Europe, the prevalence is around 21% [5], while Asia showed a higher overall prevalence of 23% [6]. Within Asia, the prevalence in Southeast Asia (37.46%) is significantly higher than that in the Western Pacific region (21.95%) [6]. In turn, sarcopenia may contribute to the onset and progression of T2DM by exacerbating glucose dysregulation and local inflammation due to reduced muscle mass and metabolic capacity [7]. Importantly, individuals with coexisting T2DM and sarcopenia exhibit a significantly higher risk of adverse outcomes such as falls, fractures, and disability, compared with those with sarcopenia alone [8, 9]. Therefore, elucidating the underlying mechanisms of T2DM complicated by sarcopenia and developing mechanism-based preventive and therapeutic strategies are of critical importance.

The human gastrointestinal tract harbors a complex and diverse ecosystem of microorganisms, collectively known as the gut microbiota, which includes bacteria, fungi, and viruses. Under normal physiological conditions, the gut microbiota maintains a complex symbiotic relationship with the host and plays a critical role in various processes, including metabolism, immune regulation, and nutrient absorption [10]. However, dysbiosis of the gut microbiota has been implicated in the onset and progression of both T2DM and sarcopenia [11, 12]. A growing body of evidence indicates that individuals with T2DM or sarcopenia often exhibit alterations in gut microbiota composition and associated metabolic dysfunction [13, 14]. Among these microbial metabolites, short-chain fatty acids (SCFAs) act as key mediators involved in various physiological processes, including reduction of inflammation, enhanced insulin sensitivity, and improvements in mitochondrial and autophagic function [15-18]. These effects suggest that SCFAs may play a pivotal regulatory role in the pathogenesis of T2DM complicated by sarcopenia. Therefore, SCFAs are emerging as promising therapeutic targets for the prevention and treatment of T2DM complicated by sarcopenia.

Exercise, recognized as a low-cost and safe non-pharmacological strategy, has demonstrated substantial benefits in patients with T2DM-associated sarcopenia by promoting muscle hypertrophy and strength, as well as improving glucose regulation, lipid profiles, and insulin responsiveness [19-22]. Moreover, exercise has been shown to positively modulate gut microbiota composition, particularly by increasing the abundance of SCFA-producing bacteria [23]. This microbial shift has been shown to lead to elevated levels of SCFAs, which may mediate the beneficial metabolic and anti-inflammatory effects of exercise [19, 24]. Given these emerging findings on exercise and SCFA modulation, this review systematically examines the relationship between SCFAs and T2DM complicated by sarcopenia, as well as the effects of exercise on SCFA production. Furthermore, we explore the potential mechanisms through which exercise-induced modulation of SCFAs may contribute to the prevention and management of T2DM complicated by sarcopenia, aiming to provide novel insights into exercise-based therapeutic strategies.

## 2. Overview of SCFAs

SCFAs refer to saturated aliphatic acids composed of one to six carbon atoms, most notably acetic, propionic, butyric, and formic acids, as well as their respective isomeric forms [25]. They are the main products of dietary fiber fermentation by gut microbiota in the colon, with daily production estimated at 500-600 mmol depending on fiber intake. Acetic, propionic, and butyric acids constitute approximately 95% of total SCFAs, while formic, valeric, and caproic acids are present in smaller amounts [26].

Following their synthesis in the intestinal lumen, SCFAs are absorbed by intestinal epithelial cells via monocarboxylate transporter 1, sodium-coupled monocarboxylate transporter 1, and the down-regulated in adenoma transporter. Within epithelial cells, SCFAs enter the tricarboxylic acid cycle to generate adenosine triphosphate, supporting epithelial integrity [27]. Unmetabolized SCFAs reach the liver via the portal circulation, contributing to gluconeogenesis, while a portion enters systemic circulation to influence peripheral tissues [28].

Beyond maintaining gut homeostasis and promoting nutrient absorption, SCFAs play key roles in regulating energy metabolism and immune function. They activate G-protein-coupled receptors (GPRs), especially GPR41 (free fatty acid receptor 3, FFAR3) and GPR43 (free fatty acid receptor 3, FFAR2), which are broadly expressed in various tissues. Activation of these receptors modulates immune responses by promoting immune cell differentiation and enhancing host defense [29, 30]. Additionally, SCFAs improve glucose and lipid metabolism, enhance glucose tolerance, and increase insulin sensitivity [31]. Of particular interest, emerging evidence suggests that SCFAs can modulate the mammalian target of rapamycin (mTOR) signaling pathway, further underscoring their involvement in systemic metabolic regulation and highlighting their potential as therapeutic targets [32].

## 3. SCFAs with T2DM complicated by sarcopenia

### 3.1 SCFAs with T2DM

Patients with T2DM exhibit marked gut microbiota dysbiosis, characterized by reduced microbial diversity, a significant decrease in SCFA-producing beneficial bacteria, and an increase in pathogenic bacteria. Qin et al. analyzed the gut microbiota composition of 345 Chinese patients with T2DM showed that moderate gut microbiota dysbiosis was prevalent in T2DM patients, as indicated by a decrease in the abundance of commonly found butyrate-producing bacteria, such as *Roseburia* and *Faecali-bacterium prausnitzii*. This dysbiosis was accompanied by an increase in the number of opportunistic pathogens, as well as the enhancement of sulfate reduction and anti-oxidative stress related metabolic pathways in intestinal microbial function [33]. Similarly, Allin et al. reported that, compared with healthy individuals, Danish adults with prediabetes exhibited significant gut microbiota abnormalities, characterized by a reduced abundance of SCFA-producing bacteria (e.g., *Clostridium spp.*) and the mucin-degrading bacteria *Akkermansia muciniphila* [34]. In addition, other important SCFA-producing bacteria, including *Akkermansia*, *Bifidobacterium*, and *Bacteroides*, were also found to be decreased in T2DM patients [35]. These microbial taxa are believed to play crucial roles in maintaining intestinal barrier integrity, regulating inflammatory responses, and supporting metabolic homeostasis [36]. Notably, SCFA levels, particularly butyrate, were significantly reduced in T2DM patients and were strongly correlated with several metabolic parameters, including body mass index, blood glucose levels, serum cholesterol, fecal bile acid concentrations, and blood lipid profiles [37, 38]. These findings suggest that SCFAs may be involved in the pathogenesis and progression of T2DM.

Inulin, a prebiotic fiber that cannot be digested or absorbed directly by the human body, can be fermented by the gut microbiota to produce SCFAs. A randomized controlled clinical trial demonstrated that inulin intake promoted SCFA production and improved lipid oxidation, thereby significantly enhancing glycemic control [39]. Another study further demonstrated that supplementation with inulin-propionate ester elevates colonic propionate levels and attenuates weight gain, primarily through the stimulation of glucagon-like peptide-1 (GLP-1) secretion [40]. Moreover, metformin, the first-line pharmacological treatment for T2DM, has also been shown to remodel the gut microbiota composition in high-fat diet-induced obese mouse models, leading to the enrichment of *Akkermansia muciniphila*, improved glucose tolerance, and reduced adipose tissue inflammation [41]. Therefore, modulating gut microbiota composition to enhance SCFA production may represent a promising therapeutic strategy for T2DM.

### 3.2 SCFAs with Sarcopenia

With aging, significant alterations occur in the gut microbiota, characterized by a reduction in microbial diversity and a decreased relative abundance of SCFA-producing bacteria. Conversely, gut microbial dysbiosis can further exacerbate sarcopenia and other age-related pathological processes [42]. Compared to healthy controls, patients with sarcopenia exhibit a markedly reduced gut microbial diversity, with an increased abundance of *Lactobacillus* and a decreased abundance of SCFA-producing genera such as *Lachnospira* and *Coprococcus* [43]. Similarly, Lee et al. reported that the abundance of *Prevotella*, an important SCFA-producing genus, was significantly lower in elderly individuals with sarcopenia. Furthermore, even in healthy elderly individuals, a significant decline in the abundance of butyrate-producing genera such as *Ruminococcus* has been observed, accompanied by an increase in opportunistic pathogens [44]. However, most studies to date have primarily focused on the compositional changes of gut microbiota, with limited direct assessment of SCFA levels in sarcopenic populations. A recent study measured fecal SCFA concentrations in aging mice, revealing that levels of acetate, butyrate, isobutyrate, valerate, and isovalerate were significantly lower in aged mice compared to young counterparts [45]. These findings suggest a close interplay among sarcopenia, gut microbiota dysbiosis, and SCFA deficiency.

Moreover, probiotic supplementation has shown considerable promise in delaying sarcopenia progression. Supplementation with *Lactobacillus plantarum* TWK10 has been reported to ameliorate gut dysbiosis, reduce the *Firmicutes*-to-*Bacteroidetes* ratio, and improve skeletal muscle mass and function in aged mice. TWK10 administration also promotes the enrichment of SCFA-producing genera such as *Ruminococcus* and *Coprococcus*, thereby enhancing SCFA levels and improving muscle metabolic status [46]. Studies conducted in elderly populations have similarly demonstrated that TWK10 supplementation increases muscle glycogen storage, augments energy supply, and enhances muscle function and endurance [47]. Additionally, Cao et al. established a murine model of sarcopenia and demonstrated that butyrate supplementation could significantly delay disease progression by activating interleukin-2 (IL-2)/IL-2-independent group 2 innate lymphoid cells (ILC2s)/basic leucine zipper ATF-like transcription factor 13 (BATF) signaling pathways [48]. Therefore, targeting gut microbiota to enhance SCFA production may represent a promising therapeutic strategy for the prevention and treatment of sarcopenia.

Taken together, both T2DM and sarcopenia are characterized by a reduced abundance of SCFA-producing bacteria and decreased SCFA levels, which may be further exacerbated in individuals with both conditions. This synergistic dysbiosis may contribute to metabolic dysregulation, chronic low-grade inflammation, and accelerated muscle degradation. Although direct evidence remains limited, these findings highlight the urgent need for future research to elucidate SCFA profiles—namely, the relative levels of key SCFAs such as acetate, propionate, and butyrate—and the composition of the gut microbiota in patients with coexisting T2DM and sarcopenia. Such insights may help identify novel targets for microbiota-based therapeutic strategies.

## 4. Exercise with SCFAs

Exercise, as an independent modulator of the gut microbiota, can effectively increase the relative abundance of SCFA-producing bacteria, thereby enhancing SCFA levels [49]. In recent years, aerobic exercise, resistance training, combined aerobic and resistance training, and high-intensity interval training (HIIT) have been the primary types of exercise interventions investigated for individuals with T2DM complicated by sarcopenia. Therefore, this review focuses on these four types of exercise interventions (Table 1).

**Table 1:** Summary of the effects of different exercise modalities on SCFA-producing microbiota and SCFA levels.

Study	Characteristics of Participants	Intervention Protocol	Intervention Effect
Lambert et al. (2015) [50]	T2DM mice	AE, 2.87 m/min, 60 min/time,3 times/week, 6 weeks	*Lactobacillus*↑ *Leuconostoc*↑
Matsumoto et al. (2008) [51]	SD rats	AE, Voluntary wheel running, 4 weeks	Butyrate↑
Motiani et al. (2020) [52]	Middle-aged patients with insulin resistance	AE, 60% VO_2max_, 5 times/week, 60 min/session, 2 weeks	*Veillonella*↑ *Faecalibacterium*↑
Wei et al. (2022) [58]	T2DM mice	RE, 10%-70% of body weight, 7 times/week, 2 groups/day, climbed the ladder 3 times/group, 8 weeks	*Ruminococcus*↑ *Roseburia*↑ Acetate↑ Butyrate↑
Liu et al. (2020) [62]	Male patients with prediabetes	AE+RE, 80%-95% HR_max_,3 times/week, 70 min/session, 12 weeks	*Lachnospiraceae*↑ Acetate↑ Propionate↑ Total SCFAs↑
Zhong et al. (2021) [63]	Older Women	AE+RE, AE: 60%-75% HR_max_, 3 times/week, 60 min/session, 8 weeks; RE: 60%-70% 1RM, 3 times/week, 60 min/session, 8weeks	*Lachnospiraceae*↑ *Mitsuokella*↑
Ma et al. (2020) [57]	T2DM mice	AE: 15 m/min, 7 times/week, 60 min/session, 8 weeks; RE: 10%-70% of body weight, 7 times/week, 2 groups/day, climbed the ladder 3 times/group, 8 weeks; AE+RE: AE and RE were each performed three times per week on separate days, 8 weeks	Acetate↑ Propionate↑ Butyrate↑
Torquati et al. (2023) [67]	People with T2DM	MICT: 55%-69% HR_max_, 4 times/week, 52.5 min/session, 8 weeks; HIIT: 3 times/week, 26 min/session (78 min/week), 8 weeks; includes 3 min aerobic warm-up at 50-60% HR_max_, 4 min high-intensity aerobic exercise at 85-95% HR_max_, 1 min rest, followed by 8×1 min high-intensity resistance exercises (RPE≥17, 5-25 reps), each with 1 min rest, ending with 3 min aerobic cool-down at 50-60% HR_max_	MICT:*Bifidobacterium*↑ *Akkermansia muciniphila*↑ Total SCFAs → HIIT: *Oscillospira*↑*Erysipelotrichales*↑ Total SCFAs →
Solouki et al. (2024) [68]	High-fat diet-induced diabetic rats	MICT: 30%-50% VO_2max_, 5 times/week, 30 min/session, 10 weeks; HIIT: 85-90%VO_2max_, 5 times/week, 5 min warm-up at 30%-40% VO_2max_, 1 min running sprint at 85-90% VO_2max_, 3 min rest, 10 weeks	*Akkermansia*↑ *Butyricicoccus*↑ Butyrate↑ Propionate↑
Han et al. (2024) [23]	T2DM rats	MICT: 17 m/min, 7 times/week, 30 min/session, 8 weeks; HIIT: 25 m/min (7min), 15 m/min (3 min), 4 goups/day,7 times/week, 8 weeks	Acetate↑ Butyrate↑

Notes: “↑”indicates a significant improvement(P<0.05); “→”indicates a no significant change; “↓”indicates a significant reduction. Abbreviations:RE, resistance exercise; 1RM, one repetition maximum; AE, aerobic exercise; RPE, rating of perceived exertion; HR_max_, maximum heart rate; VO_2max_, maximal oxygen uptake; MICT: moderate-intensity continuous training; HIIT: high-intensity interval training.

### 4.1 Aerobic exercise

Increasing preclinical evidence indicates that aerobic exercise enhances the production of SCFAs by modulating gut microbiota composition and increasing the abundance of SCFA-producing taxa. Lambert et al. demonstrated that 6 weeks of low-intensity treadmill training significantly increased the levels of *Lactobacillus* and *Leuconostoc* in the feces of T2DM mice, both of which are major SCFA producers [50]. Matsumoto et al. found that voluntary wheel running significantly elevated cecal butyrate levels in rats, providing further support for the role of aerobic exercise in enhancing SCFA production through microbial mechanisms [51].

Similarly, Han et al. reported that 8 weeks of moderate-intensity continuous training (MICT) in T2DM rats effectively modulated the gut microbiota composition, improved glucose and lipid metabolism, and significantly increased the abundance of SCFA-producing bacteria as well as fecal levels of acetate and butyrate. Metabolic pathway analysis further revealed significant upregulation of the PPW-5676 and P163-PWY pathways, both of which are closely associated with SCFA biosynthesis [23]. These findings suggest that the metabolic improvements observed in diabetic rats following aerobic exercise may be partially mediated by gut microbiota remodeling.

Clinical studies further support the beneficial effects of aerobic exercise in shaping the gut microbiota toward an SCFA-producing profile. For example, Motiani et al. demonstrated that a two-week cycling intervention in insulin-resistant middle-aged adults significantly increased the abundance of *Veillonella* and *Faecalibacterium*, which contribute to SCFAs production through lactate metabolism and butyrate synthesis, respectively [52]. In a cross-sectional study involving 100 elderly participants, Zhong et al. observed a positive correlation between standing time and the abundance of butyrate-producing taxa, including *Ruminococcaceae*, *Lachnospiraceae*, and *Bifidobacterium*, whereas sedentary time showed a negative association with *Ruminococcaceae* [53].

Moreover, multiple studies have indicated a time-dose-dependent relationship between exercise duration and gut microbiota modulation [54-56]. Longer interventions are associated with progressive increases in SCFA-producing genera such as *Bifidobacterium* and *Lactobacillus*, as well as elevated concentrations of SCFAs. Collectively, these findings highlight the critical role of aerobic exercise in maintaining gut microbial homeostasis —defined as a balanced and diverse microbial ecosystem that supports host metabolic and immune health—and enhancing SCFAs production.

### 4.2 Resistance exercise

Resistance exercise, also referred to as strength training, typically involves the use of fitness equipment such as dumbbells or resistance bands. Compared to aerobic exercise, current evidence on the effects of resistance exercise on gut microbiota composition and SCFA levels in individuals with T2DM or older adults remains limited. Ma et al. investigated the effects of resistance training on serum SCFA levels in T2DM mice using an 8-week progressive ladder-climbing protocol with tail loading (10%-70% of body weight, 7 sessions per week, 2 sets per day, 3 climbs per set). The results showed that resistance exercise significantly increased serum concentrations of acetate and butyrate [57]. In a subsequent study by the same research group, 8 weeks of resistance training led to increased relative abundances of the SCFA-producing genera *Ruminococcus* and *Roseburia* in feces, along with elevated SCFA concentrations in both serum and feces [58]. These findings suggest that resistance exercise may enhance SCFA production by promoting the proliferation of SCFA-producing gut bacteria.

However, findings from human studies have been inconsistent. McKenna et al. reported that a 10-week resistance training program combined with high protein intake significantly increased the abundance of SCFA-producing taxa such as *Veillonellaceae* and *Akkermansia* in middle-aged and older adults [59]. In contrast, Agyin-Birikorang et al. reported that a 10-week resistance training program in untrained healthy older adults improved skeletal muscle mass but did not significantly alter gut microbiota α- or β-diversity, nor did it affect SCFA levels in feces or serum [60]. These discrepancies may be due to differences in dietary control, as the latter study did not standardize dietary intake, particularly fiber, which is a major driver of SCFA production [61]. This lack of dietary standardization may partly explain the discrepancy between animal and human findings. Therefore, the specific effects of resistance exercise on SCFA-producing bacteria and SCFAs are still worth exploring.

### 4.3 Combination exercise

Combined exercise, which integrates resistance and aerobic training, is a widely adopted physical activity strategy. Liu et al. demonstrated that a 12-week intervention combining high-intensity combined exercise significantly reduced the homeostasis model assessment of IR (HOMA-IR) in men with prediabetes. This intervention also enhanced the relative abundance of SCFA-producing taxa, including *Lachnospiraceae*, and significantly increased fecal levels of acetate, propionate, and total SCFAs. Additionally, the intervention upregulated key genes involved in propionate biosynthesis, including K00245, K01754, and K13992 [62]. These findings suggest that combined exercise not only enriches SCFA-producing microbiota but also enhances the genetic potential for SCFA synthesis, thereby promoting microbial fermentation of dietary fiber in the gut.

Similarly, an 8-week combined exercise program in sedentary elderly women increased relative abundances of SCFA-producing taxa, including *Lachnospiraceae* and *Mitsuokella* [63]. Notably, Ma et al. investigated the effects of 8 weeks of aerobic, resistance, and combined exercise on SCFA levels in T2DM mice. The results showed that, compared to the control group, all exercise groups exhibited significantly increased serum concentrations of acetate, propionate, and butyrate, with the combined exercise group showing the most pronounced elevation [57].

The advantages of combined exercise in promoting SCFA production may be attributed to the integration of the distinct physiological benefits provided by aerobic and resistance exercise. Aerobic exercise enhances gut perfusion and mucosal oxygenation, whereas resistance exercise contributes to gut microbiota remodeling via elevated circulating lactate levels, which influence microbial composition and metabolic activity [64-66]. Therefore, combined exercise may constitute the most effective form of physical activity for increasing SCFA levels. However, evidence from human clinical trials remains limited and warrants further investigation.

### 4.4 High-intensity interval training

HIIT, an emerging exercise modality increasingly applied in the prevention and management of chronic diseases, is characterized by high exercise intensity, strong physiological stimulation, and short training duration. Previous studies have demonstrated that HIIT can effectively modulate gut microbiota composition and promote the production of SCFAs. However, whether HIIT provides superior benefits compared to MICT remains controversial. Torquati et al. compared gut microbiota changes in individuals with T2DM following 8 weeks of either MICT or HIIT intervention (3 sessions per week for both protocols). They found that exercise intensity selectively influenced microbial structure. Specifically, MICT significantly increased the relative abundance of typical butyrate-producing genera such as *Bifidobacterium* and *Akkermansia muciniphila*, whereas HIIT enhanced the abundance of other butyrate-producing taxa, including *Oscillospira* and members of *Erysipelotrichales*. Despite these differences in microbial composition, no significant difference was observed in total SCFA output between the two groups [67].

Interestingly, animal studies have reported inconsistent results regarding the relative efficacy of HIIT versus MICT in enhancing SCFA levels. For instance, Solouki et al. conducted a 10-week intervention (5 sessions per week) in a high-fat diet-induced diabetic rat model and found that both MICT and HIIT increased the *Firmicutes*-to-*Bacteroidetes* ratio and significantly elevated SCFA levels. Notably, HIIT showed superior effects in increasing the relative abundance of *Akkermansia* and *Butyricicoccus*, as well as in enhancing butyrate and propionate concentrations, suggesting that HIIT may have greater potential in promoting SCFA synthesis [68]. In contrast, Han et al. conducted an 8-week intervention with seven training sessions per week in a T2DM rat model, and reported that MICT exerted more favorable effects on enhancing microbial evenness and species diversity, as well as increasing acetate and butyrate concentrations [23]. These divergent results may stem from differences in baseline metabolic conditions, exercise frequency, or total training volume. Additionally, the degree of dietary control and slight inconsistencies in animal models may further contribute to these observed discrepancies.

In summary, HIIT has been shown to effectively enhance the abundance of SCFA-producing bacteria and increase SCFA levels. Nevertheless, whether HIIT is superior to MICT in this regard requires further investigation through large-scale, well-controlled human trials with standardized protocols. Moreover, current evidence regarding the effects of HIIT on SCFA production is primarily derived from studies in T2DM populations or animal models, while research specifically targeting SCFA responses to HIIT in older adults remains lacking. Given the potential differences in gut microbial composition and metabolic responsiveness in the elderly compared to other populations, further interventional or observational clinical studies are warranted to elucidate these effects in aging individuals.

## 5. Exercise with T2DM complicated by sarcopenia

With the increasing comorbidity of T2DM complicated by sarcopenia, identifying effective intervention strategies has become crucial for improving metabolic dysfunction and muscle degeneration. Among them, exercise, as a non-pharmacological intervention, has garnered growing attention due to its high safety profile, good adherence, and multi-target regulatory effects. Studies have shown that various types of exercise, including resistance exercise, aerobic exercise, combined exercise, and HIIT, exhibit distinct physiological effects and clinical potentials in glycemic control and the improvement of muscle mass and function [69, 70].

Resistance exercise is currently one of the most widely studied exercise modalities for individuals with T2DM complicated by sarcopenia, demonstrating significant clinical benefits. Chien et al. conducted a 12-week progressive sandbag resistance exercise program in 40 T2DM patients with sarcopenia. Results showed that, compared to the control group, the intervention group exhibited significant reductions in glycated hemoglobin (HbA1c) levels and glycemic variability, along with notable improvements in grip strength, muscle mass, and physical performance [71]. Similarly, Zhao et al. reported that even short-term moderate-intensity resistance exercise effectively reduced average blood glucose levels and glycemic fluctuations in T2DM patients with sarcopenia, and decreased the risk of hypoglycemic events [72]. Additionally, Brazo-Sayavera et al. conducted an 11-week intervention study during the COVID-19 pandemic to assess the impact of a multicomponent exercise program centered on resistance exercise in frail older adults with T2DM. Despite a general decline in physical activity levels during the intervention, the increase in sedentary time was significantly lower in the exercise group compared to controls, accompanied by a slight reduction in self-perceived disability and maintenance of health-related quality of life (HRQoL) [73]. These findings suggest that resistance training can offer protective benefits even under special circumstances such as a pandemic. Overall, resistance exercise not only effectively improves muscle mass, strength, and function in T2DM patients with sarcopenia but also contributes to glycemic control. Current resistance training protocols for older adults with chronic diseases typically adopt a progressive resistance training model, performed 2-3 times per week. These programs generally include at least 5 to 10 exercises targeting major muscle groups (upper body, lower body, and core), with each session lasts 20-30 minutes and consists of 10-15 repetitions per set. The initial intensity is recommended at 40%-60% of one repetition maximum (1RM), gradually increasing to 70%-85%, while avoiding excessive fatigue [74].

Although aerobic exercise is less effective than resistance training in inducing muscle hypertrophy, it plays an important role in enhancing oxidative metabolism of skeletal muscle, reducing HbA1c levels, improving IR, and increasing muscular endurance [75, 76]. It also contributes to reducing intramuscular fat deposition and improving muscle function [77]. Studies have indicated that participation in aerobic activities such as group fitness dancing can slow skeletal muscle loss in T2DM patients [78]. Animal experiments showed that six weeks of moderate-intensity aerobic training significantly reduced blood glucose and HbA1c levels in db/db mice and increased the cross-sectional area of the gastrocnemius muscle fibers. Furthermore, aerobic exercise activates key signaling pathways related to glucose and lipid metabolism and insulin sensitivity, facilitating muscle regeneration and the maintenance of metabolic homeostasis, thus offering protection against T2DM-induced sarcopenia [79]. The currently recommended aerobic exercise regimens include brisk walking, jogging, cycling, and swimming, performed more than three times per week, 30-45 minutes per session, totaling 150-300 minutes per week. The recommended intensity is moderate, corresponding to 40%-60% of maximal oxygen consumption (VO_2_max) or 65%-75% of maximal heart rate [80].

Among various exercise modalities, combined training has attracted widespread attention in recent years due to its integration of both aerobic and resistance exercise benefits. A prospective study reported that combining resistance band training with aerobic exercise effectively improved frailty status in elderly T2DM patients. Six-month follow-up data showed a reduction in frailty prevalence from 34.1% to 25.0%, and a decrease in the incidence of moderate-to-severe functional impairment from 26.2% to 21.4%, suggesting that combined exercise may enhance physical function and slow the progression of frailty [81]. Ding et al. studied 114 elderly patients with T2DM complicated by sarcopenia, and found that compared with aerobic exercise alone, adding progressive resistance exercise significantly improved fasting blood glucose, glycated albumin, and HbA1c levels, as well as total muscle mass, appendicular skeletal muscle mass, and quality of life scores [21]. These findings suggest that combined exercise may be superior to aerobic exercise alone in managing T2DM complicated by sarcopenia. The synergistic effects may be attributed to the integration of distinct physiological benefits: aerobic exercise primarily enhances cardiorespiratory fitness, insulin sensitivity, and fat oxidation, while resistance training focuses on mechanical tension to stimulate muscle protein synthesis via pathways such as mTOR, thereby improving muscle mass and strength [82]. The combination of both modalities offers complementary advantages in glycemic control and muscle function restoration. Indeed, studies have shown that combined exercise is more effective in improving glycemic indices, insulin sensitivity, and physical function [70, 83, 84]. Additionally, the variety and enjoyment associated with combined exercise may enhance patient adherence and promote long-term physical activity participation. Therefore, combined exercise may represent the optimal exercise intervention for patients with T2DM complicated by sarcopenia. Current clinical guidelines for diabetes management recommend incorporating resistance exercise at least three times per week in addition to regular aerobic exercise, with 2-3 sets of 8-10 repetitions targeting major muscle groups, performed at moderate intensity.

HIIT has been considered a potentially efficient strategy for improving metabolic health within a short period, as it elicits strong metabolic responses per unit of time, including modulation of glycolysis, aerobic metabolism, fatty acid β-oxidation, and mitochondrial biogenesis [85]. However, further research has indicated that while HIIT has positive effects on weight, waist circumference, fasting blood glucose, HOMA-IR, HbA1c, and cardiorespiratory fitness in T2DM patients, its superiority over MICT in improving these outcomes is only evident when HIIT programs are of sufficient duration, volume, and length [86, 87]. This requirement for high-frequency, high-volume HIIT may negate its originally perceived time-efficiency and simplicity. Therefore, for elderly individuals with T2DM complicated by sarcopenia, limited physical capacity and poor tolerance may hinder long-term adherence, thereby affecting the sustainability and feasibility of HIIT interventions.

In summary, studies on exercise interventions for T2DM patients with sarcopenia suggest that resistance training, aerobic exercise, and combined training all hold significant therapeutic potential. Resistance training improves muscle mass and function while contributing to glycemic control through muscle hypertrophy and strength enhancement. Aerobic exercise enhances skeletal muscle metabolism, reduces IR, and delays muscle loss. Combined training, which integrates the benefits of both modalities, is more effective in improving glycemic parameters, muscle health, and quality of life, while also promoting better adherence. Although HIIT is characterized by strong metabolic responses and high efficiency, its applicability in this population is limited due to concerns regarding intensity, training volume, and sustainability. Future intervention strategies should emphasize individualization and feasibility, optimizing exercise prescriptions by integrating the advantages of various exercise modes to achieve dual goals of glycemic control and muscle health improvement.

**Figure 1.**
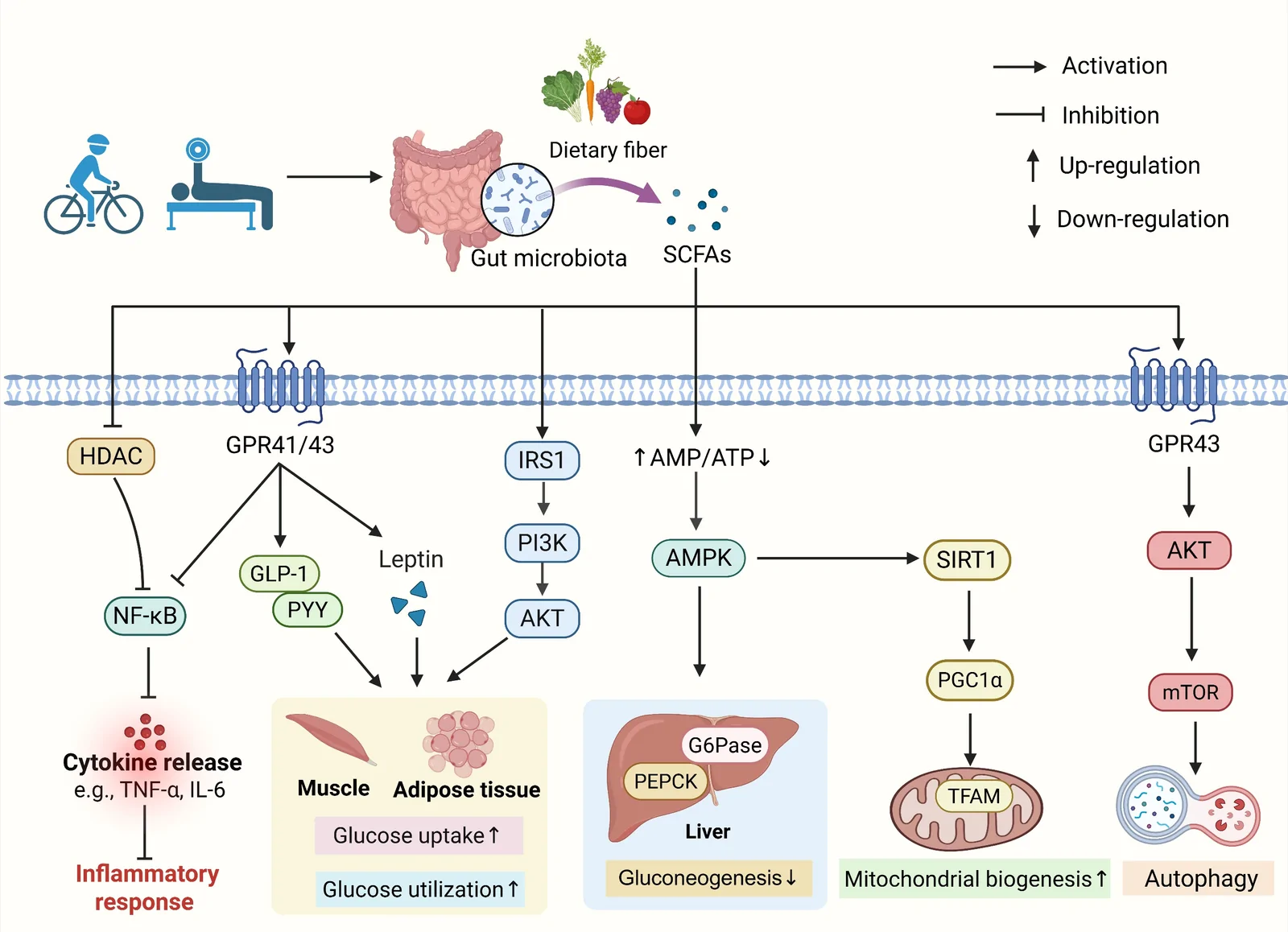
**The possible mechanism of exercise regulating SCFAs in the intervention of T2DM complicated by sarcopenia**. Created with BioRender.com. Abbreviations: SCFAs, short chain fatty acids; GPR41/43, G-protein-coupled receptors 41/43; NF-κB, nuclear factor-kappa B; TNF-α, tumor necrosis factor-α; IL-6, interleukin-6; GLP-1, glucagon-like peptide-1; PYY, peptide YY; ATP, adenosine triphosphate; AMP, adenosine monophosphate; IRS1, insulin receptor substrate 1; PI3K, phosphatidylinositol-3-kinase; AKT, protein kinase B; G6Pase, glucose-6-phosphatase; PEPCK, phosphoenolpyruvate carboxykinase; mTOR, mammalian target of rapamycin; SIRT1, sirtuin 1; PGC-1α, peroxisome proliferator-activated receptor-gamma coactivator 1;TFAM, mitochondrial transcription factor A.

## 6. Mechanisms of Exercise-Regulated SCFAs in the Prevention and Treatment of T2DM complicated by sarcopenia

The pathophysiological mechanisms of T2DM complicated by sarcopenia are multifactorial and remain incompletely elucidated. Current evidence indicates that chronic low-grade inflammation, impaired autophagy, mitochondrial dysfunction, and insulin resistance may act synergistically to drive disease progression [2, 88, 89]. Exercise-induced elevations in SCFA production have been shown to attenuate inflammation, enhance insulin sensitivity, regulate autophagic activity, and improve mitochondrial function, thereby playing a crucial role in the prevention and management of T2DM complicated by sarcopenia (Fig. 1). The following sections elaborate on these mechanisms.

### 6.1 Reduced inflammatory response

In individuals with T2DM, chronic hyperglycemia, insulin resistance, and glucotoxicity collectively contribute to a pro-inflammatory metabolic environment [90]. This state is marked by elevated circulating levels of inflammatory cytokines, including tumor necrosis factor-α (TNF-α), interleukin-6 (IL-6), interleukin-1β (IL-1β), and C-reactive protein (CRP), all of which are known to accelerate muscle protein breakdown (MPB) and impair regenerative capacity [91]. Persistent low-grade inflammation promotes MPB by activating the ubiquitin-proteasome system (UPS) and the autophagy-lysosome pathway, while concurrently inhibiting key anabolic signaling cascades, particularly the phosphoinositide 3-kinase (PI3K)/protein kinase B (Akt)/mTOR pathway [92].

Emerging evidence highlights the crucial anti-inflammatory role of gut microbiota-derived SCFAs in this context. SCFAs exert systemic effects by modulating GPRs, such as GPR41, GPR43, and GPR109A, and by inhibiting inflammatory pathways, including the nuclear factor-kappa B (NF-κB) signaling cascade [93]. However, T2DM is commonly associated with gut dysbiosis and a reduced abundance of SCFA-producing microbial taxa, which further amplifies systemic inflammation and contributes to skeletal muscle wasting [94]. Therefore, chronic inflammation represents a central patho-physiological link between metabolic dysregulation and muscle degeneration in T2DM-induced sarcopenia. Targeting this inflammatory axis—particularly through microbiota-directed interventions aimed at restoring SCFA production—may offer a promising therapeutic strategy to prevent or attenuate muscle loss in individuals with T2DM.

Exercise was shown to modulate gut microbiota composition and enhance SCFA production, thereby exerting anti-inflammatory effects. Vijay et al. reported that a 6-week exercise intervention in elderly individuals significantly elevated butyrate levels and decreased serum concentrations of pro-inflammatory markers such as TNF-α and IL-6 [95]. Similarly, Solouki et al. demonstrated that 10 weeks of HIIT increased the abundance of *Akkermansia* and *Butyrivibrio*, elevated SCFA levels (including butyrate and propionate) in the cecum, downregulated cecal Toll-like receptor 4 (TLR4) expression, and reduced serum IL-6 levels [68]. Furthermore, a study involving voluntary wheel running in mice revealed increased fecal abundance of *Lactobacillus* and *Eubacterium nodatum*, elevated mRNA expression of GPR109A and GPR41 in the colon, and enhanced concentrations of SCFAs (e.g., acetate, propionate, and isobutyrate), accompanied by reduced expression of inflammation-related markers [96].

Supplementation with acetate has also been shown to activate GPR43 expression in the mouse colon and significantly reduce LPS-induced TNF-α secretion [97]. Moreover, treatment with propionate and butyrate in LPS-pretreated neutrophils inhibited histone deacetylases (HDACs), thereby suppressing NF-κB activation and attenuating the inflammatory response [98]. These findings suggest that SCFAs exert anti-inflammatory effects through dual mechanisms: by activating GPR41/GPR43, and by inhibiting HDACs, both of which converge on the suppression of NF-κB signaling.

In summary, exercise-induced production of SCFAs by the gut microbiota may alleviate chronic low-grade inflammation by directly activating GPR41/GPR43 receptors on the cell membrane or by passively diffusing into cells to inhibit HDACs and subsequently suppress NF-κB signaling. Thus, exercise interventions may help prevent and manage T2DM with sarcopenia through SCFAs-mediated anti-inflammatory pathways.

### 6.2 Improvement of insulin resistance

In T2DM, insulin sensitivity of peripheral tissues—particularly skeletal muscle—is markedly diminished, accompanied by downregulation of IGF-1, resulting in suppression of the PI3K/Akt/mTOR signaling pathway [99]. Consequently, glucose uptake is impaired, hyperglycemia persists, and MPS is inhibited. Moreover, abnormal intermuscular fat deposition is commonly observed in the skeletal muscle of individuals with T2DM, which activates the forkhead box O (FoxO) family of transcription factors. This in turn promotes the activation of the UPS, upregulating the expression of muscle-specific atrophy-related genes such as muscle atrophy Fbox (MAFbx)/Atrogin-1 and muscle-specific RING-finger protein 1 (MuRF-1), thereby accelerating MPB and leading to muscle mass loss [100]. Therefore, IR can disrupt systemic glucose homeostasis and insulin sensitivity, disturb the balance between muscle protein synthesis (MPS) and MPB, and ultimately lead to sarcopenia.

SCFAs play a critical role in glucose regulation and glycogen metabolism, serving as important mediators in maintaining host glucose homeostasis. SCFAs are produced by gut microbiota via the fermentation of dietary fiber and can activate G protein-coupled receptors GPR41 and GPR43 on intestinal L-cells, thereby stimulating the secretion of GLP-1 and peptide YY (PYY) [101, 102]. GLP-1 enhances insulin secretion via activation of GLP-1 receptors on pancreatic β-cells, while PYY facilitates glucose uptake and utilization in skeletal muscle and adipose tissue, thereby improving peripheral insulin sensitivity [103]. Indeed, in 3T3-L1 adipocytes and C2C12 myotubes, SCFAs have been shown to increase basal glucose uptake and enhance insulin sensitivity by activating GPR41 [104].

In addition, SCFAs modulate key genes in the insulin signaling pathway through epigenetic mechanisms. For instance, butyrate inhibits HDAC activity and increases histone H3 lysine 9 acetylation, thereby upregulating transcriptional expression of insulin receptor substrate-1 (IRS1) [105]. Following insulin stimulation, tyrosine-phosphorylated IRS1 recruits the p85 regulatory subunit of PI3K, leading to activation of AKT [106]. Activation of AKT facilitates the translocation of GLUT4 to the plasma membrane, thereby enhancing glucose uptake in skeletal muscle and adipose tissues. This mechanism contributes to improved glycemic control and increased insulin responsiveness [107].

Furthermore, SCFAs activate cellular energy sensors such as AMP-activated protein kinase (AMPK) by increasing the intracellular AMP/ATP ratio [108]. In skeletal muscle, AMPK activation suppresses glycogen synthesis while promoting glucose uptake and fatty acid oxidation [109]. In the liver, AMPK inhibits gluconeogenesis by downregulating key enzymes including glucose-6-phosphatase and phosphoenol-pyruvate carboxykinase [110]. Sakakibara et al. reported that dietary supplementation with 0.3% acetic acid for 8 weeks in diabetic mice significantly upregulated hepatic AMPK expression and suppressed the expression of gluconeogenesis-related genes, thereby reducing fasting blood glucose levels [111].

In addition to the direct regulation of blood glucose, recent studies have shown that SCFAs are also involved in the process of leptin secretion. Sanz-Martos et al. demonstrated that exogenous SCFA supplementation in mice promoted leptin secretion via GPR41/GPR43 activation, thereby enhancing hepatic glycogen synthesis and skeletal muscle glucose uptake [112].

In conclusion, SCFAs can alleviate IR by regulating multiple molecular signaling pathways. Exercise promotes SCFAs production by the gut microbiota. These SCFAs enhance insulin sensitivity by activating GPR41/43-mediated intestinal hormone release, inhibiting HDACs, and regulating the IRS1-PI3K-AKT-GLUT4 pathway. Moreover, SCFAs activate AMPK signaling, thereby suppressing hepatic gluconeogenesis and promoting fatty acid oxidation. SCFAs may also enhance metabolic regulation via leptin signaling. These mechanisms collectively enhance insulin signaling and peripheral tissue sensitivity to insulin, thereby contributing to the maintenance of MPS/MPB homeostasis and mitigating the development or progression of T2DM with sarcopenia. Therefore, as an important metabolite of exercise-mediated metabolic health benefits, SCFAs can play an important role in the prevention and treatment of T2DM with sarcopenia by improving IR.

### 6.3 Regulate autophagy in cells

Autophagy is an evolutionarily conserved metabolic process in eukaryotic cells that plays a critical role in maintaining cellular homeostasis by eliminating excess or damaged cellular components and harmful substances. It is closely associated with IR [113]. In individuals with T2DM, autophagic function is often impaired, as evidenced by decreased expression of autophagy-related proteins such as microtubule-associated protein 1 light chain 3 (LC3), autophagy-related gene 7 (Atg7), Unc-51-like kinase 1 (Ulk1), and Beclin-1, leading to reduced autophagic flux and accumulation of misfolded proteins, thereby promoting skeletal muscle atrophy [114]. Activation of multiple signaling pathways, such as the PI3K/Akt pathway, myostatin signaling, the UPS, and the autophagy-lysosomal system, can effectively enhance autophagic activity and delay skeletal muscle loss [115]. Furthermore, Atg7-deficient mice exhibit markedly increased oxidative stress, accompanied by activation of the p38 MAPK and JNK signaling pathways, resulting in reduced pancreatic β-cell numbers and impaired insulin secretion, thereby exacerbating IR [116]. Similarly, in Ulk1-knockout mice, autophagy flux is significantly reduced, with concomitant mitochondrial DNA damage and elevated ROS levels, ultimately leading to mitochondrial dysfunction and muscle weakness [117]. Studies have also demonstrated that the expression of mitophagy-related proteins such as Bnip3 and Parkin declines in T2DM, contributing to the pathological accumulation of damaged mitochondria in skeletal muscle [118]. However, Romanello et al. reported that overexpression of Bnip3 can also trigger mitochondrial fragmentation, activate autophagy, and induce muscle atrophy [119]. Thus, it is evident that dysregulation of autophagy under T2DM conditions contributes to skeletal muscle atrophy to varying degrees.

Recently, the regulatory role of SCFAs in autophagy has garnered increasing attention. The mechanistic target of mTOR signaling pathway, a central regulator of autophagy, has been implicated in SCFA-mediated modulation of aging-related autophagy. Liu et al. found that SCFA supplementation in aged SAMP8 mice activated the AKT/mTOR signaling pathway in skeletal muscle and improved muscle mass, strength, and function [120]. Tang et al. demonstrated that butyrate supplementation significantly upregulated GPR43 receptor expression in the skeletal muscle of db/db mice. This was associated with activation of the PI3K/Akt/mTOR signaling pathway, inhibition of autophagy, and amelioration of muscle atrophy [121]. Notably, inhibition of GPR43 signaling abolished the beneficial effects of butyrate, suggesting that butyrate regulates the PI3K/Akt/mTOR pathway via GPR43 to modulate autophagy, thereby preventing or mitigating T2DM complicated by sarcopenia [121].

Interestingly, Qiao et al. observed that sodium butyrate treatment of mouse small intestinal endocrine STC-1 cells activated the PI3K/Akt/mTOR pathway and simultaneously increased LC3B-II expression, indicating that butyrate may also promote autophagy under certain conditions [122]. In a murine model of Duchenne muscular dystrophy (DMD), Kalkan et al. found that sodium butyrate supplementation not only restored muscle strength and alleviated autophagic impairment in vivo, but also enhanced autophagic flux in vitro in LPS-stimulated C2C12 myoblasts through activation of the GPR109A receptor [123]. These findings suggest that the regulation of autophagy by SCFAs is not unidirectional; rather, it involves a finely orchestrated modulation of autophagic homeostasis. This modulation may depend on tissue-specific context, baseline autophagic activity, the underlying disease type, and its stage of progression (Table 2).

**Table 2:** Dual Role of SCFAs in the Regulation of Autophagy under Different Conditions.

Study	SCFA Type	Cell/Tissue Context	Pathological Condition	Effect on Autophagy	Mechanism
Liu et al. (2024) [120]	SCFAs	Skeletal muscle	Aging	Inhibition	AKT/mTOR↑
Tang et al. (2022) [121]	Butyrate	Skeletal muscle	T2DM rodent model	Inhibition	GPR43/PI3K/AKT/m TOR↑
Qiao et al. (2020) [122]	Butyrate	STC-1 cells	Parkinson's disease	Activation	PI3K/AKT/mTOR↓
Kallan et al. (2023) [123]	Butyrate	C2C12 myotubes	DMD	Activation	GPR109A↑

Notes: “↑”indicates a significant improvement(P<0.05); “↓”indicates a significant reduction. Abbreviations: SCFAs, short chain fatty acids; AKT, protein kinase B; mTOR, mechanistic target of rapamycin; PI3K, phosphatidylinositol 3-kinase; GPR43, G-protein-coupled receptor 43; GPR109A, G-protein-coupled receptor 109A; T2DM, type 2 diabetes mellitus; DMD, Duchenne muscular dystrophy.

Exercise exerts bidirectional modulation of autophagy: for pathological states characterized by insufficient or excessive autophagy, exercise can restore autophagy levels toward homeostasis, thereby ameliorating or delaying disease progression. For instance, Lee et al. demonstrated that the LC3II/LC3I protein ratio was significantly elevated in the skeletal muscle of a diabetic muscle atrophy model compared to controls. Four weeks of swimming training normalized the expression of autophagy-related proteins and significantly increased skeletal muscle mass, suggesting that exercise can improve T2DM with sarcopenia by attenuating excessive autophagic activity [124]. He et al. reported that an 8-week treadmill exercise intervention increased the LC3II/LC3I ratio and Beclin-1 expression while decreasing p62 protein levels in skeletal muscle, thereby improving glucose intolerance induced by a high-fat diet in mice [125]. This indicates that exercise-induced enhancement of skeletal muscle autophagy contributes to improved metabolic health.

The ability of exercise to bidirectionally regulate autophagy may involve SCFAs as key mediators. Yang et al. found that in a T2DM animal model, exercise increased the abundance of *Bacteroides* and their SCFA metabolites, upregulated the expression of autophagy-related proteins in skeletal muscle, and improved IR. Targeted inhibition of GPR43 abrogated the beneficial effects of exercise on IR in vivo and acetate-mediated effects in vitro, suggesting that exercise-enhanced SCFA production may promote autophagy in skeletal muscle through GPR43 signaling, thereby ameliorating IR [126]. Although no studies have directly demonstrated that exercise-mediated modulation of SCFAs prevents or treats T2DM complicated by sarcopenia through the attenuation of excessive autophagy, existing evidence strongly supports a critical role for SCFAs in maintaining autophagy homeostasis. Thus, exercise may correct autophagy dysfunction by modulating SCFA levels, thereby preventing and treating T2DM complicated by sarcopenia.

### 6.4 Improve mitochondrial dysfunction

Elderly patients with T2DM complicated by sarcopenia are often in a state of chronic hyperglycemia, which leads to increased production of ROS and elevated oxidative stress levels, thereby inducing oxidative damage to mitochondrial DNA (mtDNA) and subsequently causing mitochondrial dysfunction [127]. Meanwhile, ROS can regulate NF-κB and FoxO transcription factors, activate the UPS and the autophagy-lysosome pathway, resulting in impaired mitochondrial biofunction, increased apoptosis, and autophagy defects, thus accelerating skeletal muscle atrophy [128]. In addition, AMPK can induce mitochondrial biogenesis in skeletal muscle by activating peroxisome proliferator-activated receptor-γ coactivator-1α (PGC-1α), thereby enhancing cellular energy metabolism [129, 130]. However, in individuals with T2DM, the PGC-1α-nuclear respiratory factor 1 (NRF1)-mitochondrial transcription factor A (TFAM) signaling pathway becomes impaired, leading to reduced mitochondrial function and quantity, which in turn promotes the onset and progression of sarcopenia [131]. Moreover, gut microbiota dysbiosis can damage mitochondrial ultrastructure, such as causing mitochondrial swelling and cristae disruption, thereby inducing mitochondrial metabolic disorders and epithelial cell apoptosis, which further exacerbates mitochondrial dysfunction, contributing to glucose and lipid metabolism disturbances and skeletal muscle atrophy [132]. Therefore, targeting gut microbiota regulation to improve mitochondrial dysfunction may serve as a potential strategy for the prevention and treatment of T2DM complicated by sarcopenia.

Current evidence suggests that exercise intervention can modulate gut microbiota composition, increase the production of SCFAs, enhance mitochondrial quantity in skeletal muscle, improve mitochondrial energy supply efficiency, and ameliorate glucose and lipid metabolism, ultimately helping to alleviate metabolic syndrome. Liu et al. conducted a 12-week HIIT intervention in men with prediabetes and found that exercise significantly increased the abundance of SCFA-producing bacteria such as *Bacteroides* and *Clostridiales*, elevated SCFA levels, improved glucose metabolism disorders, and enhanced insulin sensitivity [62]. Further research by Ye et al. demonstrated that SCFAs modulated the gut microbiota composition in rats with diabetic nephropathy, activate the AMPK/SIRT1/PGC-1α signaling pathway, and enhance mitochondrial function to regulate systemic energy metabolism [133]. Meanwhile, Walsh et al. demonstrated that the gut microbiota-derived metabolite butyrate can upregulate mitochondrial porin and TFAM levels, enhancing oxidative metabolism capacity in skeletal muscle and delaying muscle aging [134].

Therefore, exercise may target the modulation of gut microbiota composition, increase SCFA levels, activate the AMPK/SIRT1/PGC-1α/TFAM signaling pathway, and promote mitochondrial biogenesis and functional restoration, thereby delaying the onset and progression of T2DM complicated by sarcopenia. However, direct evidence confirming that exercise improves mitochondrial dysfunction and prevents T2DM complicated by sarcopenia specifically through regulating gut microbiota-derived SCFAs is currently lacking. Thus, future studies are needed to further elucidate and validate this mechanistic link.

## 7. Conclusions and perspectives

T2DM complicated by sarcopenia represents a major clinical challenge due to its multifactorial pathogenesis and the bidirectional interplay between metabolic and muscle dysfunction. Emerging evidence suggests that SCFAs, as key microbial metabolites linking gut microbiota and host physiology, may serve as crucial mediators in exercise-induced improvements in glucose metabolism and muscle health. This review highlights that various exercise modalities—particularly combined training—can modulate gut microbiota composition, increase the abundance of SCFA-producing bacteria, and elevate circulating SCFA levels. These changes may improve insulin sensitivity, mitochondrial function, inflammation, autophagy, and muscle anabolism via multiple molecular pathways, such as AMPK activation, GPR41/43 signaling, and HDAC inhibition.

Despite these promising findings, several knowledge gaps remain. While some evidence exists, most human studies to date have not systematically evaluated SCFA levels, and the impact of specific exercise types—particularly resistance training—on SCFA production remains underexplored. The optimal exercise prescription (i.e., intensity, duration, and frequency) for maximizing gut microbiota modulation and SCFA production remains to be determined. Moreover, most existing trials are limited by small sample sizes, short follow-up durations, and insufficient control of confounding variables such as dietary intake, antibiotic use, and baseline gut microbiota composition.

In addition, few studies have focused specifically on older adults, who may exhibit distinct gut microbial characteristics and metabolic responses. These limitations highlight the urgent need for well-designed, large-scale randomized controlled trials with rigorous dietary control, standardized exercise protocols, and age-specific considerations to better elucidate the role of SCFAs in mediating exercise-induced benefits in the elderly population.

Moreover, it is unclear which specific SCFA-producing microbes or individual SCFAs confer the most pronounced metabolic and musculoskeletal benefits in the context of exercise intervention. Given the strong modulatory influence of diet on gut microbiota, exercise alone may be insufficient to elicit maximal therapeutic effects. Future research should explore integrative approaches—such as combining exercise with dietary fiber intake or probiotic supplementation—to synergistically enhance SCFA production and its downstream biological effects.

Additionally, the mechanistic axis linking exercise, SCFAs, and the management of T2DM complicated by sarcopenia remains insufficiently characterized. Currently, the most available evidence regarding the effects of SCFAs on muscle metabolism and systemic inflammation is derived from animal studies or in vitro models. However, mechanistic investigations specifically focusing on T2DM or aging-related skeletal muscle alterations—particularly those involving critical signaling pathways such as GPRs and PI3K/Akt/mTOR—are notably insufficient. Importantly, interspecies differences in gut microbiota composition, immune responses, and skeletal muscle metabolic regulation may limit the translational relevance of these preclinical findings derived from animal or in vitro models to human physiology. Therefore, a deeper understanding of SCFA-mediated mechanisms is essential—not only to improve clinical outcomes in this high-risk population but also to inform the development of personalized, microbiota-targeted exercise interventions that can effectively address this complex comorbidity of T2DM with sarcopenia.

## Data Availability

Data availability is not applicable to this article as no new data were created or analyzed in this study.
